# Comparison of Problem-based Learning With Lecture-based Learning

**DOI:** 10.5812/ircmj.5186

**Published:** 2014-05-05

**Authors:** Parisa Khoshnevisasl, Mansour Sadeghzadeh, Saeidah Mazloomzadeh, Reza Hashemi Feshareki, Akefeh Ahmadiafshar

**Affiliations:** 1 Zanjan Social Determinants of Health Research Center, Zanjan University of Medical Sciences, Zanjan, IR Iran; 2 Zanjan Community Oriented Medical Education, Zanjan University of Medical Sciences, Zanjan, IR Iran; 3 Zanjan Metabolic Disease Research Center, Zanjan University of Medical Sciences, Zanjan, IR Iran

**Keywords:** Education, Lecture, Student, Medical, Problem-based Learning

## Abstract

**Background:**

Problem-based learning (PBL) is one of the most commonly used educational methods in medical schools of different countries. By working through this method, students think critically, generate ideas, and acquire the knowledge and skills required to become a doctor.

**Objectives:**

This study aimed to compare problem-based learning with lecture-based learning in the education of medical students.

**Materials and Methods:**

This crossover interventional study was conducted on 40 medical students in pediatric ward of Zanjan University of Medical Sciences. All of the students were enrolled in the study and divided into two groups by simple randomization. Then two topics in pediatric courses were chosen. One of the topics was presented as LBL for the first group and as PBL for the second group. The other topic was presented as PBL for the first group and as LBL for the second group.

**Results:**

The median score of the exam was higher in the intervention group compare to the control group for both topics. However, the difference was not statistically significant. Students preferred problem-based learning over lecture-based learning because of motivation boost, a higher quality of education, knowledge retention, class attractiveness, and practical use.

**Conclusions:**

Students’ knowledge was similar in both methods.

## 1. Background

Problem-based learning (PBL) is one of the most commonly used educational methods in medical schools. In this method, students use scenarios to define their own learning objectives. The success of PBL depends on the quality of the scenarios (1). Presenting clinical problems is the starting point for learning in PBL. By working through these problems, students think critically about the nature of the problem, generate ideas, and acquire the knowledge and skills required to become a doctor (2). It seems that students will have a better knowledge retention with this method (1), and PBL increases in-depth training, and helps students to perform better in examinations (3). Although supporters of PBL state that learning motivation is one of the benefits of this method, some mention that it is time-consuming and does not provide a better clinical competence (4). In LBL method, students solely receive information from the lecturer and attempt to memorize the content instead of understanding the concepts and using them. Therefore, at the patient’s bedside, they unconsciously and merely satisfy themselves with the routine work, deal passively with new situations, and make no effort toward thinking and innovation to diagnose and meet the existing requirements (5). Many studies were conducted to compare PBL with the traditional LBL. With respect to acquiring knowledge, investigations showed different results. In some studies, PBL did not show any preference over LBL on the trainees' knowledge (6-11). On the other hand; many studies showed that students got better scores in PBL method (3, 12-15).

## 2. Objectives

To clarify the impact of new teaching methods on our students’ knowledge and evaluate their satisfaction, we conducted the present study to compare PBL with LBL in Zanjan University of Medical Sciences.

## 3. Materials and Methods

In this crossover study, all fifth-year medical students (40), who introduced to the pediatric ward of Zanjan University of Medical Sciences from October 2010 to March 2011, were enrolled. They were divided into two groups by simple randomization. The first group was trained in pediatric ward in Ayatollah Moussavi Hospital (group A), and the second group (group B) was introduced to the clinics and emergency room. Six weeks later, the groups exchanged their training center.

Two common pediatric topics, which are not taught in pediatric theory classes or at the patient's bedside, were chosen (syncope and speech delay). For the first group of students (A), the "syncope" topic (a) was taught as lecture by the researcher in the pediatric ward. The second group of students (B) learned the same topic (a) in clinics with PBL method. After 6 weeks, the , "speech delay" topic (b), was presented by PBL method to the first group (A), and the same topic (b) was taught as lecture for the second group (B). Both lectures were presented by the same lecturer.

Study design based on educational goals was determined by the consultation of three pediatricians. For the intervention group referred to clinics; the selected problem was expressed, and the scenario was prepared by trainers. Teaching methods, goals and the learning needs of the students were mentioned, and the group was referred to the sources of information without any limitation. At the beginning of each session, case documents, including patient complaints, presenting illness, and sample questions about the etiology, diagnosis, paraclinical tests and treatment were given to the students.

At the next meetings, the group presented their solutions and the students discussed and debated various aspects of the case. After some discussion, paraclinical documents such as ECG, X-ray, lab results, and patients’ photographs (if required) were presented to the students. The students discussed the problem and explained its causes, differential diagnosis and their approach to the problem.

Finally, they created a diagnostic algorithm. A sense of competition and also cooperation was created and reinforced among students. The role of the tutor was to facilitate the training process. Each group wrote their solutions and in each session, one member of each group was chosen to present the response of the group. The students discussed the solutions, and the best answers were emphasized.

At the end of the pediatric courses, and after the completion of PBL in both groups, an exam was taken to evaluate the students' knowledge, and then a satisfaction questionnaire was completed by the students. The exam tests consisted of 10 questions; each subject had almost five similar questions. In each topic, two questions were asked with the first taxonomy, two questions with the second taxonomy, and one question with the third taxonomy. The total score in each topic was 5.

The satisfaction questionnaire assessed and compared students’ opinions about PBL and LBL in the fields of learning quality, knowledge retention, practical usefulness, class attractiveness, answering to exams, motivation to study, and students’ preferences.

The validity of the questionnaire was assessed in the previous studies (16). Furthermore, the validity of the exam questions was ascertained by the content validity determined by experts and pediatric academics’ opinions. After data collection, the statistical analysis was performed by Mann-Whitney U test using SPSS 16.0 software. Differences were considered to be statistically significant if the P value was less than 0.05. This study has been approved by the ethics committee of Zanjan University of Medical Sciences (901372).

## 4. Results

Forty students (25 females, 62.5% and 15 males, 37.5%) entered into this study during a 6-month period. After performing the crossover trial in both subjects (syncope and speech delay), two tests with different questions, but quite similar in structure were held at the end. In the intervention group (PBL) for "syncope", the range of scores was between 2 and 5. The median score was 3.5 (3 - 4). In the control group in which "syncope" was presented by LBL, the highest score was 5, and the lowest one was zero. The median score of students in this group was 3 (2 - 4). Although the lowest score in the control group was less than the lowest one in the intervention group, the median score was not statistically different between two groups (P = 0.5).

Regarding the intervention group who attended "speech delay" as PBL, the range of scores was between 1 and 5. The median score of students in this group was 3 (2 - 4). In the control group in which "speech delay" was pre sented as LBL, the highest score was 5, and the lowest was zero. The median score of students in the control group was 4 (3 - 4). Likewise, although the lowest score in the control group was less than the lowest in the intervention group, the median difference between two groups was not significant (P = 0.7).

**Figure 1.**
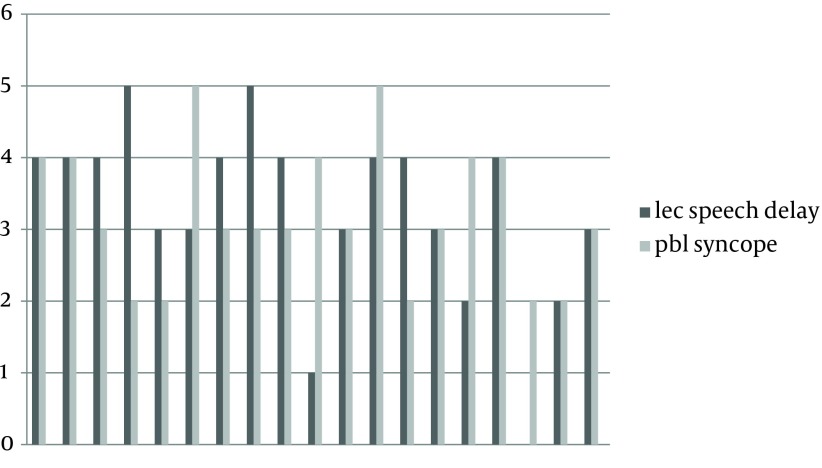
Each Student’s Scores in the Intervention for Syncope (PBL) and (LBL) for Speech Delay (Control)

**Figure 2.**
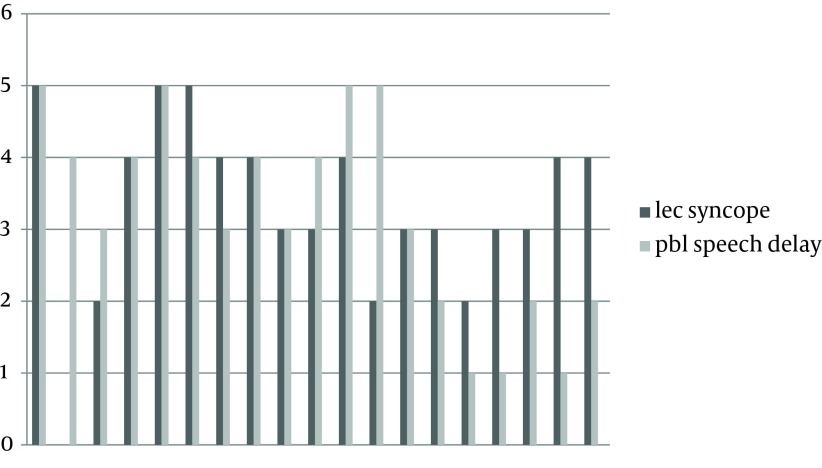
Each Student’s Scores in the Intervention for Speech Delay (PBL) and (LBL) for Syncope (Control)

Table 1 shows the median scores in the control and intervention groups in both topics. The students’ scores in each group have been shown in Figures 1 and Figures 2. A total of 25 students filled out a questionnaire about their satisfaction regarding PBL and LBL. The results have been shown in Table 2.

**Table 1.** Median (25 – 75th percentile) Scores for Control and Intervention Groups

Variable	Intervention	Control	P value
**Syncope**	3.5 (3 - 4)	3 (2 - 4)	0.5
**Speech delay**	3 (2 - 4)	4 (3 - 4)	0.7

**Table 2.** Students’ Satisfaction of Teaching Methods ^a, b^

-	LBL	PBL	No Difference
**Learning quality**	3 (12)	18 (72)	4 (16)
**Knowledge retention**	5 (20)	15 (60)	5 (20)
**Practical usefulness**	5 (20)	17 (68)	3 (12)
**Class attractiveness**	6 (24)	17 (68)	2 (8)
**Answering to exams**	14 (56)	7 (28)	4 (16)
**Motivation to study**	1 (4)	22 (88)	1 (4)
**Student’s suggestion**	5 (20)	17 (68)	3 (12)

^a^ Data are presented in No. (%).

^b^ Abbreviation: PBL, problem-based learning; LBL, lecture-based learning.

## 5. Discussion

In the present study, students preferred PBL because of motivation boost, quality learning, knowledge retention, class attractiveness, and practical usefulness of contents. However, in the case of answering the exam questions, lecture method was considered more effective, presumably because of the speaker's emphasis on teaching key points.

In our study, we did not find a significant difference in students' scores between control and intervention groups for both topics (syncope and speech delay); however, the minimum score in the control group was lower. Considering the design of our study (crossover trial), the factors affecting student scores such as individual characteristics, IQ, memory, and motivation have been largely eliminated. Furthermore, given that the tutor of intervention group and the speaker on both topics has been the same person, confounding factors related to the lecturer, such as knowledge, manner of expression, art of teaching, and emphasizing key points, have been omitted. Besides, topics were selected from the subjects that were not taught by any other teacher in theory classes or at the patient's bedside to reduce the effect of confounding factors.

The students were periodically introduced to the clinics every six weeks. First, it seemed that the last group of the students, who held PBL sessions in the upcoming weeks to exams, should be more prepared to answer the exam questions, and we could not find a solution to prevent that preparedness. However, we were surprised to find out that the results of their exams did not differ much.

With respect to the knowledge, investigations have yielded different results. Smits PB conducted a study in a postgraduate medical training program concerning the management of mental health problems for occupational health physicians in The Netherlands. He showed that in both PBL and LBL groups, knowledge had equally increased right after the programs and decreased equally after the follow-up. He concluded that problem-based program appeared to be more effective than the lecture-based program in improving performance. Both programs, however, were equally effective in improving knowledge levels (6).

In a study on 52 graduated anesthesiologists, attended in Professional and Continuing Education courses in the topic of air embolism, carried on by Carrero, PBL was compared with LBL. The knowledge of participants was assessed before and after the intervention; no significant differences were observed in the area of immediate knowledge before and after the intervention (7). The study of Goodyear on 14 senior house officers in Birmingham showed that, learning outcomes were similar in PBL and LBL (8). Khan conducted a study on fourth- and fifth-year medical students in Pakistan, to compare the effect of PBL versus LBL on the knowledge and attitude of students. He found that both groups demonstrated a similar level of knowledge (9).

Johnston in Hong Kong, undertook a randomized-controlled trial to compare PBL with LBL, and concluded that PBL was less effective at imparting knowledge than customary LBL (10). The study of Choi on 90 Korean nursing students to compare PBL with LBL revealed that learning outcomes of problem based learning were not statistically different from LBL, although students in PBL group showed improved abilities in problem solving, self-directed learning and critical thinking (11). On the other hand, McParland et al. compared PBL with LBL in the field of psychiatry in England. He concluded that performance of the students holding PBL was better in both multiple-choice questions and the viva. But there were no differences between the two methods in the learning style and attitude of students (3). In the study of Tack on dental students in The Netherlands, students’ knowledge turned out to be higher in the topic chosen for PBL (12). In a study conducted by Lin in Taiwan on nursing students, the group who received PBL as the training method showed more satisfaction, critical thinking and self-motivated learning. And it was shown that PBL training was more effective than conventional teaching (13).

In the study of Moreno Lopez, who carried out on dental students in Bologna, PBL participants obtained higher scores compared with the LBL group. PBL participants spent more time on group work and literature analysis (14). The results of the study of Anyaehie in Nigeria indicated that PBL increased students’ attendance, participation in classes and performance in examination (15). Hwang in an investigation to compare PBL with LBL in cardiorespiratory section of nursing courses in Chicago determined that the level of knowledge in the PBL group was significantly higher than that of students in the LBL group (17). Meo assessed knowledge and skills of undergraduate medical students in a respiratory physiology course and concluded that students in PBL group obtained significantly higher scores compared to LBL approach (18).

Considering students’ satisfaction, many studies showed that students prefer PBL (5, 12, 17, 19, 20). However, in the investigation of Smits, the PBL group was less satisfied with the course. This was the only study that we found with a different conclusion on students’ satisfaction (6).

Certainly, several studies have been conducted to compare the two methods, which had different results. This could be due to the fact that the intervention and control groups, as well as the lecturer and tutor, were not identical. Or the way to carry out the problem-based learning method and the interval time to the exam were different.

The advantage of this study was its crossover design, which could prevent intersubject variability. For a more accurate comparison of these two methods with regard to knowledge retention, further studies are recommended.

## References

[1] Wood DF (2003). ABC of learning and teaching in medicine Problem based learning.. BMJ..

[2] Onyon C (2012). Problem-based learning: a review of the educational and psychological theory.. Clin Teach..

[3] McParland M, Noble LM, Livingston G (2004). The effectiveness of problem-based learning compared to traditional teaching in undergraduate psychiatry.. Med Educ..

[4] Kilroy DA (2004). Problem based learning.. Emerg Med J..

[5] Dehkordi AH, Heydarnejad MS (2008). The impact of problem-based learning and lecturing on the behavior and attitudes of Iranian nursing students. A randomised controlled trial.. Dan Med Bull..

[6] Smits PB, de Buisonje CD, Verbeek JH, van Dijk FJ, Metz JC, ten Cate OJ (2003). Problem-based learning versus lecture-based learning in postgraduate medical education.. Scand J Work Environ Health..

[7] Carrero EJ, Gomar C, Fábregas N, Penzo W, Castillo J, Villalonga A (2008). Clase magistral versus aprendizaje basado en caso/problema para la enseñanza del embolismo aéreo en formación médica continuada.. Revista Española de Anestesiología y Reanimación..

[8] Goodyear HM (2005). Problem based learning in a junior doctor teaching programme.. Arch Dis Child..

[9] Khan H, Taqui AM, Khawaja MR, Fatmi Z (2007). Problem-based versus conventional curricula: influence on knowledge and attitudes of medical students towards health research.. PLoS One..

[10] Johnston JM, Schooling CM, Leung GM (2009). A randomised-controlled trial of two educational modes for undergraduate evidence-based medicine learning in Asia.. BMC Med Educ..

[11] Choi E, Lindquist R, Song Y (2014). Effects of problem-based learning vs. traditional lecture on Korean nursing students' critical thinking, problem-solving, and self-directed learning.. Nurse Educ Today..

[12] Tack CJ, Plasschaert AJ (2006). Student evaluation of a problem-oriented module of clinical medicine within a revised dental curriculum.. Eur J Dent Educ..

[13] Lin CF, Lu MS, Chung CC, Yang CM (2010). A comparison of problem-based learning and conventional teaching in nursing ethics education.. Nurs Ethics..

[14] Moreno-Lopez LA, Somacarrera-Perez ML, Diaz-Rodriguez MM, Campo-Trapero J, Cano-Sanchez J (2009). Problem-based learning versus lectures: comparison of academic results and time devoted by teachers in a course on Dentistry in Special Patients.. Med Oral Patol Oral Cir Bucal..

[15] Anyaehie US (2007). Comparative evaluation of active learning and the traditional lectures in physiology: a case study of 200 level medical laboratory students of Imo State University, Owerri.. Niger J Physiol Sci..

[16] Mousaai Fard M, Amini K (2010). Comparison of Two Teaching Methods (Lecturing and PBL) from the Point of Zanjan Medical University Nursing Student View.. EDC J..

[17] Hwang SY, Kim MJ (2006). A comparison of problem-based learning and lecture-based learning in an adult health nursing course.. Nurse Educ Today..

[18] Meo SA (2013). Evaluating learning among undergraduate medical students in schools with traditional and problem-based curricula.. Adv Physiol Educ..

[19] Kawai Y, Yazaki T, Matsumaru Y, Senzaki K, Asai H, Imamichi Y (2007). [Comparative analysis of learning effect for students who experienced both lecture-based learning and problem-based learning in a complete denture course].. Nihon Hotetsu Shika Gakkai Zasshi..

[20] Tsou KI, Cho SL, Lin CS, Sy LB, Yang LK, Chou TY (2009). Short-Term Outcomes Of A Near-Full PBL Curriculum In A New Taiwan Medical School.. TKaohsiung J Med Sci..

